# *Gjb2* V37M Modifies Metabolic Responses to Chronic High-Fat Feeding in Mice

**DOI:** 10.3390/ijms27198603

**Published:** 2026-09-25

**Authors:** Cheng-Yu Hsieh, Ying-Chang Lu, Chuan-Jen Hsu, Tien-Chen Liu, Wei-Shiung Yang, Chen-Chi Wu, Juen-Haur Hwang

**Affiliations:** 1 Department of Otolaryngology-Head and Neck Surgery, Taichung Tzu Chi Hospital, Buddhist Tzu Chi Medical Foundation, Taichung 427213, Taiwan; chengyu384650@gmail.com (C.-Y.H.); chuanjenhsu99@gmail.com (C.-J.H.); 2 School of Medicine, Tzu Chi University, Hualien 970374, Taiwan; 3 Graduate Institute of Clinical Medicine, National Taiwan University College of Medicine, Taipei 100233, Taiwan; wsyang@ntu.edu.tw; 4 Department of Otolaryngology, National Taiwan University Hospital, Taipei 100225, Taiwan; lu16889@gmail.com (Y.-C.L.); liuent@ntu.edu.tw (T.-C.L.); 5 Department of Otolaryngology-Head and Neck Surgery, National Taiwan University Hospital Hsin-Chu Branch, Hsinchu 300195, Taiwan; 6 Department of Otolaryngology-Head and Neck Surgery, Dalin Tzu Chi Hospital, Buddhist Tzu Chi Medical Foundation, Chiayi 62247, Taiwan; 7 Department of Medical Research, China Medical University Hospital, China Medical University, Taichung 40447, Taiwan

**Keywords:** *Gjb2*, connexin 26, high-fat diet, obesity, glucose metabolism, lipid metabolism

## Abstract

GJB2 encodes connexin 26 (Cx26), and pathogenic GJB2 variants are among the most common genetic causes of hereditary hearing loss; because Cx26 is expressed in metabolically relevant tissues including liver, pancreas, and adipose tissue, whether the hearing-loss-associated c.109G>A (p.V37M) variant also modifies systemic metabolic responses to high-fat diet (HFD) exposure remains unclear. Three-month-old male wild-type (WT) and homozygous Gjb2 V37M mice were assigned to low-fat diet (LFD) or high-fat diet (HFD) groups, generating four study groups: WT-LFD, WT-HFD, V37M-LFD, and V37M-HFD. The mice were followed until 12 months of age, with dietary intervention continued for 9 months. Body weight, body mass index (BMI), fasting blood glucose, serum triglycerides, and HDL cholesterol were assessed at 3, 6, 9, and 12 months. An oral glucose tolerance test was performed at 9 months, and omental fat was measured at 12 months. WT-HFD mice showed marked progressive increases in body weight and BMI, whereas increases were less pronounced in V37M-HFD mice. At 12 months, body weight and BMI did not differ significantly between V37M-LFD and V37M-HFD mice. Fasting glucose, glucose tolerance, triglyceride, and HDL cholesterol profiles differed among groups. Omental fat weight also differed significantly, although the only significant post hoc comparison was between WT-HFD and V37M-LFD mice. These findings indicate that *Gjb2* V37M is associated with distinct weight, glycemic, and lipid responses during chronic dietary exposure, suggesting that *GJB2* variants may modify systemic metabolic adaptation to chronic high-fat feeding.

## 1. Introduction

Obesity and metabolic syndrome are major global public health issues. C57BL/6J mice are widely used to study diet-induced obesity because long-term high-fat diet (HFD) feeding increases adiposity, hyperglycemia, hyperinsulinemia, glucose intolerance, and insulin resistance [1,2]. Animal studies have shown that the proportion of dietary energy derived from fat is positively correlated with body fat accumulation [3]. Compared with animals fed low-fat diets, both lean and genetically obese mice fed HFDs exhibit greater energy intake and body weight gain [4]. However, genetic background can substantially modify metabolic responses to HFD and other dietary challenges [5]. Recent longitudinal studies also demonstrate marked heterogeneity in metabolic responses to prolonged high-fat dietary exposure [6]. In some animal models, increased adiposity occurs even in the absence of marked hyperphagia, further suggesting that genetic factors influence the metabolic response to dietary fat intake [3].

Several obesity-associated genes are involved in lipid metabolism, inflammation, insulin signaling, and adipocyte differentiation. Peroxisome proliferator-activated receptor gamma (PPAR-γ) is a key regulator of adipogenesis and obesity-related metabolism, and partial PPAR-γ deficiency can reduce HFD-induced insulin resistance [7,8]. In contrast, LDL receptor-deficient mice show greater susceptibility to HFD, partly because of impaired compensatory beta-cell function and increased beta-cell apoptosis [2]. Excess fatty acid exposure can also promote lipotoxicity and mitochondrial dysfunction in metabolically active tissues [9,10]. These findings indicate that specific genetic alterations can either attenuate or exacerbate the metabolic consequences of long-term HFD exposure.

Connexins are membrane proteins involved in intercellular communication and are also linked to metabolic regulation [11,12]. Cx26, Cx32, and Cx36 participate in pancreatic development, whereas Cx36 and Cx43 contribute to the regulation of insulin secretion [11,13,14]. Connexin signaling is also associated with adipogenesis, brown adipose tissue function, food intake, cholesterol homeostasis, and cellular stress responses [15,16,17,18,19,20]. In the liver, gap-junction signaling has also been implicated in steatohepatitis and fibrosis [21]. These observations suggest that connexin signaling may influence systemic metabolic responses.

*Gjb2* encodes connexin 26 (Cx26), which is expressed in several tissues, including the cochlea, pancreas, liver, skin, and brain [22]. Connexin 26 has been linked to hepatic cholesterol-related gene expression [18]. In humans, the *GJB2* c.109G>A (p.V37I) variant has been associated with congenital and progressive hearing loss [23]. Recently, we established and characterized a *Gjb2* c.109G>A (p.V37M) knock-in mouse model, demonstrating that it faithfully recapitulates the channel dysfunction and progressive auditory phenotype observed in humans [24], while other *Gjb2* knock-in and partial-loss mouse models also develop progressive auditory abnormalities [25,26], consistent with the established role of connexin 26 in hereditary hearing loss [27,28]. Reduced cochlear connexin 26 expression has also been reported in experimental aging models [29]. Clinical and animal studies have linked obesity-related metabolic changes to auditory dysfunction [30,31], and diet-induced obesity can alter cochlear protein kinase C signaling, energy metabolism, transcription, and autophagy [32]. However, it remains unknown whether hearing-loss-associated Gjb2 variants exert beneficial, detrimental, or neutral effects on obesity-related metabolic dysfunction. In particular, whether the Gjb2 c.109G>A (p.V37M) variant modifies systemic metabolic responses to chronic HFD exposure has not been established. Clarifying this relationship may help define whether connexin 26-related genetic variation contributes to metabolic adaptation beyond the auditory system. We therefore examined body weight, body mass index, fasting glucose, oral glucose tolerance, serum triglycerides, HDL cholesterol, and omental fat in wild-type (WT) and homozygous Gjb2 V37M mice during chronic HFD feeding.

## 2. Results

### 2.1. Body Weight Responses to HFD Differed Between WT and V37M Mice

The overall study design is shown in Figure 1A. Male WT and homozygous Gjb2 V37M mice were assigned to LFD or HFD beginning at 3 months of age and followed until 12 months.

Body weight differed significantly among the four groups at 3 months (*p* = 0.0005) and at 6, 9, and 12 months (all *p* < 0.0001; Figure 1B). Before dietary intervention, both V37M groups had numerically higher body weight than the WT groups, although not all pairwise comparisons reached statistical significance.

At 6 months, HFD-fed mice had higher body weight than their corresponding LFD controls in both genotypes. Thereafter, WT-HFD mice showed the greatest weight gain and were significantly heavier than V37M-HFD mice at 9 and 12 months (both *p* < 0.0001). In contrast, no significant difference was observed between V37M-LFD and V37M-HFD mice at either 9 or 12 months. Genotype × diet interactions were significant at 6, 9, and 12 months (all *p* < 0.0001), indicating that the effect of HFD on body weight differed between WT and V37M mice after dietary intervention (Table 1).

### 2.2. BMI Responses to HFD Differed Between WT and V37M Mice

BMI differed significantly among the four groups at all examined ages (all *p* < 0.0001; Figure 1C). Before dietary intervention, both V37M groups had significantly higher BMI values than the WT groups, whereas no significant diet-related difference was observed within either genotype. During follow-up, WT-HFD mice showed the highest BMI. BMI was significantly higher in WT-HFD than in V37M-HFD mice at 6, 9, and 12 months (all *p* < 0.0001). Both V37M groups maintained higher BMI values than WT-LFD mice, but no significant difference was observed between V37M-LFD and V37M-HFD mice at 9 or 12 months. Similarly, significant genotype × diet interactions were observed for BMI at 6, 9, and 12 months (all *p* < 0.0001; Table 1).

### 2.3. Serum Triglyceride and HDL Cholesterol Levels Differed Among Groups

Serum triglyceride levels differed significantly among groups at 3 months (*p* < 0.0001), 6 months (*p* = 0.0081), 9 months (*p* = 0.0011), and 12 months (*p* = 0.0027; Table 2). At baseline, both V37M groups had higher mean triglyceride levels than the WT groups, and group differences remained present throughout follow-up.

HDL cholesterol levels also differed significantly among groups at 3 months (*p* < 0.0001), 6 months (*p* = 0.0059), 9 months (*p* = 0.0003), and 12 months (*p* < 0.0001; Table 2). At baseline, both V37M groups had higher mean HDL cholesterol levels than the WT groups. During follow-up, the highest mean HDL cholesterol levels were observed in WT-HFD mice at 9 and 12 months. Genotype × diet interactions for triglycerides and HDL cholesterol were not uniformly significant across all time points (Table 1). For triglycerides, the interaction was significant at 6 months (*p* = 0.0007) and 12 months (*p* = 0.0063), but not at 9 months (*p* = 0.670). For HDL cholesterol, the interaction was significant at 9 months (*p* = 0.018) and 12 months (*p* = 0.043), with a borderline result at 6 months (*p* = 0.062). Notably, at the 3-month pre-intervention baseline—before dietary allocation—HDL cholesterol already differed between the groups later assigned to LFD versus HFD in a pattern resembling the subsequent genotype × diet interaction (mean difference +12.8 mg/dL, 95% CI 5.24 to 20.36, *p* = 0.0010), indicating incomplete baseline balance for this outcome despite random group assignment. This pre-existing difference should be considered when interpreting the HDL interaction observed during the HFD intervention period, and it may partly account for the borderline result at 6 months.

### 2.4. Fasting Glucose Showed Age-Specific Differences Among Groups

Fasting glucose differed significantly among groups at 3 months (*p* = 0.0389) and at 6, 9, and 12 months (all *p* < 0.0001; Figure 2). At baseline, only the comparison between WT-HFD and V37M-HFD mice reached statistical significance. During follow-up, WT-HFD mice generally showed the highest fasting glucose levels. Fasting glucose was significantly higher in WT-HFD than in V37M-HFD mice at 6, 9, and 12 months (all *p* < 0.0001). At 9 and 12 months, both V37M groups had lower fasting glucose levels than the WT groups, whereas no significant difference was observed between V37M-LFD and V37M-HFD mice. A significant genotype × diet interaction was observed at 9 months (*p* < 0.0001), whereas the interactions at 6 and 12 months were not significant (Table 1).

### 2.5. OGTT Showed Significant Group, Time, and Interaction Effects

At 9 months, blood glucose levels changed significantly across post-glucose time points during the oral glucose tolerance test (OGTT) (time effect, *p* < 0.0001; Figure 3A). Significant overall differences were also observed among the four groups (group effect, *p* < 0.0001). In addition, the group-by-time interaction was significant (*p* < 0.0001), indicating that glucose responses over time differed among the groups.

WT-HFD mice showed the highest mean blood glucose concentrations at 0, 30, 60, and 120 min. Compared with WT-HFD mice, V37M-HFD mice showed lower glucose concentrations throughout the test, with the largest differences observed at 30, 60, and 120 min. V37M-LFD mice showed a relatively high glucose peak at 30 min, followed by decreases at 60 and 120 min.

The glucose area under the curve from 0 to 120 min (AUC0–120) also differed significantly among the four groups (one-way ANOVA, F(3,36) = 47.15, *p* < 0.0001; Figure 3B). WT-HFD mice had a significantly greater AUC0–120 than the other three groups. In particular, AUC0–120 was significantly greater in WT-HFD than in V37M-HFD mice (*p* < 0.0001). V37M-LFD mice also had a greater AUC0–120 than WT-LFD and V37M-HFD mice, whereas no significant difference was observed between WT-LFD and V37M-HFD mice.

### 2.6. Omental Fat Weight Differed Among Groups at 12 Months

At 12 months, mean omental fat weights were 1.43 ± 0.46 g in WT-LFD mice, 1.77 ± 0.33 g in WT-HFD mice, 1.23 ± 0.66 g in V37M-LFD mice, and 1.59 ± 0.69 g in V37M-HFD mice (Figure 4). The overall difference among the four groups was statistically significant (one-way ANOVA, *p* = 0.0210). Tukey’s post hoc analysis showed that omental fat weight was significantly higher in WT-HFD mice than in V37M-LFD mice (*p* = 0.0145). No other pairwise comparison reached statistical significance.

## 3. Discussion

### 3.1. Gjb2 V37M Attenuates HFD-Induced Weight Gain and BMI Elevation

To our knowledge, this is the first study to examine how the *Gjb2* V37M variant influences metabolic responses to chronic HFD exposure in mice. WT-HFD mice showed marked increases in body weight and BMI, whereas these increases were attenuated in V37M-HFD mice, indicating that this hearing-loss-associated variant attenuates, rather than exacerbates, obesity-related weight gain and BMI elevation under chronic dietary fat exposure. Thus, the principal effect observed in this study was an attenuation of HFD-associated weight gain and BMI elevation rather than a generalized improvement across all metabolic outcomes.

Previous animal studies have shown that obesity can alter cochlear protein kinase C signaling, energy metabolism, transcriptional activity, and autophagy [32], indicating that the cochlea is metabolically responsive to systemic dietary stress. Our findings extend this concept in the opposite direction, suggesting that a cochlea-related gene variant may also influence systemic responses to chronic dietary stress, consistent with a potential bidirectional relationship between cochlear and systemic metabolic regulation. Interestingly, V37M mice did not show a generally lean phenotype. At several time points, V37M-LFD mice had higher body weight or BMI than WT-LFD mice. However, the additional increase associated with HFD feeding was less marked in V37M mice than in WT mice. This pattern suggests that the V37M variant may modify the response to HFD rather than simply reduce body weight under all dietary conditions. The effect of genotype therefore appears to depend partly on the dietary environment.

The omental fat results provided additional information on fat accumulation. Omental fat weight differed significantly among the four groups at 12 months, and the highest mean value was observed in WT-HFD mice. However, post hoc analysis showed a significant difference only between WT-HFD and V37M-LFD mice. No significant differences were observed between the WT-HFD and V37M-HFD mice. Therefore, the omental fat findings should be interpreted cautiously and do not clearly demonstrate that V37M reduces visceral fat accumulation.

The body-weight findings were not completely reflected by the omental fat measurements. Although V37M-HFD mice showed lower body weight and BMI than WT-HFD mice, omental fat weight did not differ significantly between these two groups. This suggests that the differences in body weight may not be explained by omental fat alone. Other fat depots, lean body mass, food intake, or energy use may also have contributed to the observed phenotype.

### 3.2. Gjb2 V37M Is Associated with a Distinct Lipid Profile

Serum triglyceride and HDL cholesterol levels also differed among groups throughout follow-up. Notably, lipid differences were already present at 3 months before dietary intervention, particularly in the V37M groups. During follow-up, the patterns of triglyceride and HDL cholesterol did not consistently parallel the changes in body weight and BMI. Connexin 26 has previously been linked to hepatic cholesterol-related gene expression [18], while broader gap-junction signaling has been implicated in liver metabolic homeostasis and injury [19,20,21]. These findings suggest that the V37M variant may be associated with a distinct lipid profile, although the present data do not support a simple protective effect on lipid metabolism. Because total cholesterol, LDL cholesterol, hepatic lipid accumulation, and body composition were not measured, the biological significance of these lipid differences remains uncertain.

### 3.3. Gjb2 V37M Is Associated with a Distinct Glycemic Phenotype

The fasting glucose and OGTT profiles suggest that *Gjb2* V37M may influence glucose regulation in addition to body weight and BMI. WT-HFD mice showed the highest fasting glucose levels during follow-up, whereas both V37M groups showed lower fasting glucose levels at 9 and 12 months. These findings indicate that the V37M background was associated with a different age- and diet-related glucose pattern. However, fasting glucose levels in WT-LFD mice were relatively high at several follow-up time points. In addition, fasting glucose values obtained from the full cohort at 9 months differed from the baseline values measured in the OGTT subset. Although the OGTT animals were randomly selected, these differences may reflect sampling variability, procedural differences, or stress related to blood collection. Therefore, the fasting glucose findings should be interpreted cautiously.

The OGTT at 9 months showed significant effects of group, time, and the group-by-time interaction, indicating that the four groups differed not only in overall glucose levels but also in how glucose levels changed after oral glucose administration. WT-HFD mice showed the highest glucose levels throughout the test. Notably, V37M-LFD mice showed higher glucose levels than V37M-HFD mice at 30 and 60 min. Consistent with the glucose curves, the OGTT AUC was also higher in V37M-LFD mice than in V37M-HFD mice.

This unexpected pattern suggests that the lower glucose response in V37M-HFD mice cannot be explained by the absence of HFD exposure. It may reflect pre-existing differences in glucose regulation between the V37M groups or differences in insulin secretion after glucose loading. Because insulin levels and insulin sensitivity were not measured, the mechanism underlying this finding remains unclear. Therefore, the glucose findings should not be interpreted as improved glucose tolerance in V37M-HFD mice. Overall, these results suggest that *Gjb2* V37M is associated with a distinct pattern of glucose regulation.

### 3.4. A Potential Role for Connexin Signaling in the Metabolic Phenotype

Connexin signaling may provide a biological explanation for the metabolic phenotype observed in this study. Cx26, Cx32, and Cx36 are involved in pancreatic development and maturation [13], whereas Cx36 and Cx43 contribute to the regulation of insulin secretion [11,14]. Gap junction signaling has also been linked to adipogenesis [15], brown adipose tissue metabolism [16], food intake [17], cholesterol homeostasis [18], and the intercellular transmission of endoplasmic reticulum stress [19,20]. Recent mouse studies further show that Cx43-dependent signaling can regulate adiposity, energy expenditure, and body-weight responses during HFD exposure [33].

These findings support the possibility that altered *Gjb2* function may change the systemic response to chronic dietary stress. The lower HFD-related weight gain and different glucose profiles observed in V37M mice may be related to changes in food intake, energy use, fat storage, or glucose metabolism. However, these mechanisms were not directly examined in the current study. Therefore, the current findings show an association between *Gjb2* V37M and a distinct metabolic phenotype, but do not prove a specific mechanism.

The present findings also suggest that the effects of Gjb2 V37M may become more apparent during prolonged dietary exposure [6]. The differences between WT-HFD and V37M-HFD mice increased during follow-up, particularly for body weight, BMI, and fasting glucose. Longitudinal HFD studies have likewise demonstrated substantial heterogeneity in metabolic responses during prolonged dietary exposure [6]. This time-dependent pattern suggests that the metabolic effect of the variant may develop gradually rather than being fully present before HFD exposure. Further mechanistic studies at multiple time points may therefore help clarify the metabolic consequences of altered Gjb2 function.

### 3.5. Implications and Limitations

The present findings suggest that the biological effects of *Gjb2* V37M may extend beyond the auditory system and influence systemic metabolic responses. The different responses of WT and V37M mice to chronic HFD exposure further indicate that the effect of the variant may depend on the dietary environment and may become more apparent with aging and prolonged metabolic stress. These findings provide a basis for future studies investigating the broader role of connexin 26 in body-weight regulation, glucose homeostasis, and lipid metabolism.

Several limitations should be acknowledged. First, only male mice were included, and sex-specific effects therefore remain unknown. Second, detailed metabolic phenotyping, including energy intake, indirect calorimetry, insulin dynamics, and multi-depot tissue analysis, was not performed. Tissue-specific Gjb2/Cx26 expression and signaling in the liver, adipose tissue, and pancreas were also not directly examined. Third, baseline variation in fasting glucose and cohort attrition during follow-up may have influenced some comparisons. Further mechanistic studies will be needed to clarify the pathways underlying the observed phenotype. Another limitation is the approximately 15-h overnight fasting period used before fasting glucose, serum triglyceride and HDL cholesterol measurements, and the OGTT. Fasting protocols in mouse metabolic studies have historically varied considerably, and overnight fasting has been commonly used. However, prolonged fasting represents a substantial metabolic challenge in mice because of their high metabolic rate and nocturnal feeding pattern. More recent studies suggest that shorter fasting periods, generally around 4–6 h, may reduce fasting-induced metabolic stress and provide a more physiological assessment of glucose and lipid metabolism. Fu et al. further showed that fasting duration can influence both glucose homeostasis and serum lipid profiles in low-fat- and high-fat-diet-fed mice [34], while shorter fasting protocols have also been recommended or used in murine metabolic and lipid studies [35,36,37]. Accordingly, the glucose and lipid findings in the present study should be interpreted in the context of the prolonged fasting protocol, which may limit direct extrapolation to human fasting physiology.

## 4. Materials and Methods

### 4.1. Animals and Dietary Intervention

Mice were housed under standard laboratory conditions at 22 ± 2 °C and 50–60% relative humidity, with a 12-h light/12-h dark cycle. Animals were housed in standard polycarbonate cages (approximately 500 cm^2^ floor area), with 4–5 mice per cage and appropriate bedding and nesting material for environmental enrichment. Water and the assigned diets were provided ad libitum throughout the study. Three-month-old male B6 mice (hereafter WT mice) and B6-background mice carrying the Gjb2 c.109G>A (p.V37M) variant (hereafter V37M mice) were randomly assigned to four groups: WT-LFD, WT-HFD, V37M-LFD, and V37M-HFD. Each group initially included 40 mice. WT-LFD and V37M-LFD mice were fed a standard rodent diet containing ≥4.5% crude fat by weight, corresponding to approximately 12–13% of total energy from fat (Fwusow Industry Co., Ltd., Taiwan), whereas WT-HFD and V37M-HFD mice were fed a purified high-fat diet providing 60% of total calories from fat (D12492, Research Diets, Inc., New Brunswick, NJ, USA). The principal ingredients and nutritional characteristics of the two diets are summarized in Table 3. Because the two diets differed in ingredient composition as well as fat content, these differences should be considered when interpreting the dietary comparisons [38]. The dietary intervention began at 3 months of age and continued for 9 months, until the mice reached 12 months of age. Measurements obtained at 3 months were considered baseline values and were collected before initiation of the dietary intervention.

### 4.2. Animal Follow-Up and Sample Selection

A total of 40 mice were initially included in each group. The number of animals decreased during follow-up because of natural death, study-related mortality, and technical failures. The numbers of mice available in the WT-LFD, WT-HFD, V37M-LFD, and V37M-HFD groups were 40/40/40/40 at 3 months, 39/38/40/39 at 6 months, 37/36/38/37 at 9 months, and 35/34/36/33 at 12 months, respectively. At 9 months, 10 surviving mice were randomly selected from each group for the OGTT and remained in the study until 12 months. At 12 months, all surviving animals were euthanized. Twenty mice from each group were randomly selected for omental fat collection and measurement. Post-mortem examination was not routinely performed for animals that died before the scheduled study endpoint; therefore, the specific causes of death were not determined.

### 4.3. Body Weight and Body Mass Index

Body weight and body length were measured at 3, 6, 9, and 12 months of age. Body length was measured manually from the tip of the nose to the base of the tail (nose-to-anus length), excluding the tail. BMI was calculated as body weight in grams divided by the square of body length in centimeters: BMI = body weight (g)/body length^2^ (cm^2^) [39].

### 4.4. Serum Triglyceride and HDL Cholesterol Measurements

Blood samples were collected at 3, 6, 9, and 12 months of age after an approximately 15-h overnight fast, with unrestricted access to water. Serum triglyceride and high-density lipoprotein cholesterol levels were measured using standard biochemical methods and expressed as mg/dL.

### 4.5. Fasting Blood Glucose and Oral Glucose Tolerance Test

For fasting blood glucose measurements, food was removed at 18:00 on the preceding day, and blood samples were collected at approximately 09:00 the following morning after an overnight fast of approximately 15 h. Blood glucose was measured using blood obtained from the tail tip.

At 9 months of age, 10 mice were randomly selected from each group for the oral glucose tolerance test (OGTT). The same overnight fasting protocol was used. Mice were administered glucose (2 g/kg body weight) via oral gavage. Mice were not anesthetized during the OGTT. Blood glucose levels were measured from tail-tip blood before glucose administration and at 30, 60, and 120 min thereafter using a portable glucose meter.

The area under the glucose concentration–time curve from 0 to 120 min (AUC0–120) was calculated using the trapezoidal rule in GraphPad Prism based on glucose measurements obtained at 0, 30, 60, and 120 min.

### 4.6. Omental Fat Measurement

At 12 months of age, all surviving mice were euthanized. Twenty mice were randomly selected from the surviving animals in each group for omental fat measurements. Omental fat was collected and weighed, and the results were expressed in grams.

### 4.7. Ethical Approval

The animal study protocol was approved by the Institutional Animal Care and Use Committee of Dalin Tzu Chi Hospital (IACUC No. 1001012-01; approved on 3 November 2011). The generation and maintenance of the Gjb2 V37M mouse model were conducted in accordance with animal welfare guidelines approved by the National Taiwan University College of Medicine and College of Public Health Institutional Animal Care and Use Committee (Approval No. 20120513; approved on 20 May 2013).

### 4.8. Statistical Analysis

Data are presented as mean ± standard deviation (SD) in tables and as mean ± standard error of the mean (SEM) in line graphs, unless otherwise specified. Omental fat data are presented as individual points with mean ± SD. Differences in body weight, BMI, fasting blood glucose, serum triglycerides, and HDL cholesterol among the four groups at each age were analyzed using one-way ANOVA followed by Tukey’s multiple-comparisons test. The 9-month OGTT data were analyzed using two-way repeated-measures ANOVA, with group as the between-subject factor and post-glucose time as the within-subject factor. The group-by-time interaction was used to determine whether the glucose responses differed among the groups over time. One-way ANOVA followed by Tukey’s multiple-comparisons test was used to compare the four groups at each OGTT time point. The glucose area under the curve from 0 to 120 min was calculated using the trapezoidal method. Differences in AUC among the four groups were analyzed using one-way ANOVA followed by Tukey’s multiple-comparisons test. Omental fat weight at 12 months was analyzed using one-way ANOVA followed by Tukey’s multiple-comparisons test. In addition, 2 × 2 factorial analyses were performed at each post-intervention time point to evaluate genotype × diet interactions. Interaction effects were reported with corresponding 95% confidence intervals and *p*-values. All statistical tests were two-sided, and a *p*-value < 0.05 was considered statistically significant. Statistical analyses were performed using STATA version 10.0 (StataCorp, College Station, TX, USA) and GraphPad Prism version 10.0.

### 4.9. Generative AI Use

Generative artificial intelligence was used only for language editing, wording refinement, and preparation of a schematic mouse illustration in Figure 1A.

## 5. Conclusions

In conclusion, *Gjb2* V37M was associated with attenuated HFD-induced increases in body weight and BMI, together with distinct fasting glucose and OGTT profiles. Omental fat weight also differed among groups, although the pairwise differences were limited. These findings suggest that *Gjb2* V37M may modify systemic metabolic responses to chronic HFD exposure, broadening the possible biological role of *Gjb2* beyond the auditory system and providing a basis for further investigation of connexin-related metabolic regulation.

## Figures and Tables

**Figure 1:**
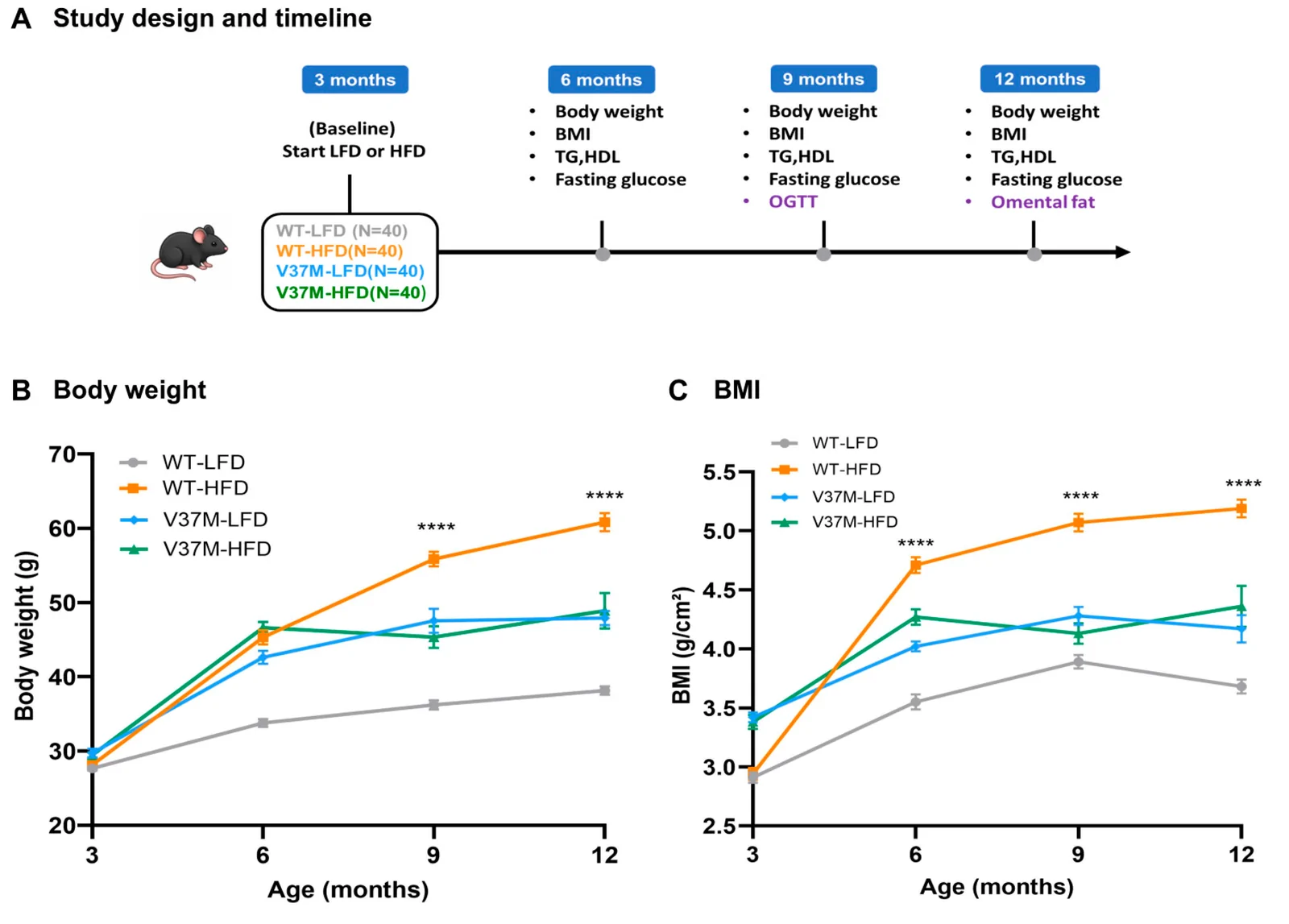
Study design and longitudinal changes in body weight and BMI in WT and *Gjb2* V37M mice under LFD or HFD. (**A**) Overview of the experimental design. Male WT and homozygous *Gjb2* V37M mice were assigned to a low-fat diet (LFD) or high-fat diet (HFD) beginning at 3 months of age. Body weight, body mass index (BMI), triglycerides (TGs), high-density lipoprotein cholesterol (HDL), and fasting blood glucose were assessed longitudinally, with oral glucose tolerance testing (OGTT) performed at 9 months and omental fat collected at 12 months. (**B**) Longitudinal changes in body weight at 3, 6, 9, and 12 months. (**C**) Longitudinal changes in BMI at the same time points. Measurements at 3 months were obtained before initiation of the dietary intervention. Data are presented as mean ± SEM. Differences among the four groups at each age were analyzed using one-way ANOVA followed by Tukey’s multiple-comparisons test. For body weight, overall group differences were significant at 3 months (*p* = 0.0005) and at 6, 9, and 12 months (all *p* < 0.0001). For BMI, overall group differences were significant at all examined ages (all *p* < 0.0001). Asterisks indicate the comparison between WT-HFD and V37M-HFD mice at the indicated time points (**** *p* < 0.0001). Sample sizes for WT-LFD, WT-HFD, V37M-LFD, and V37M-HFD were 40/40/40/40 at 3 months, 39/38/40/39 at 6 months, 37/36/38/37 at 9 months, and 35/34/36/33 at 12 months. Abbreviations: WT, wild-type; LFD, low-fat diet; HFD, high-fat diet; BMI, body mass index; TG, triglycerides; HDL, high-density lipoprotein cholesterol; OGTT, oral glucose tolerance test; SEM, standard error of the mean.

**Figure 2:**
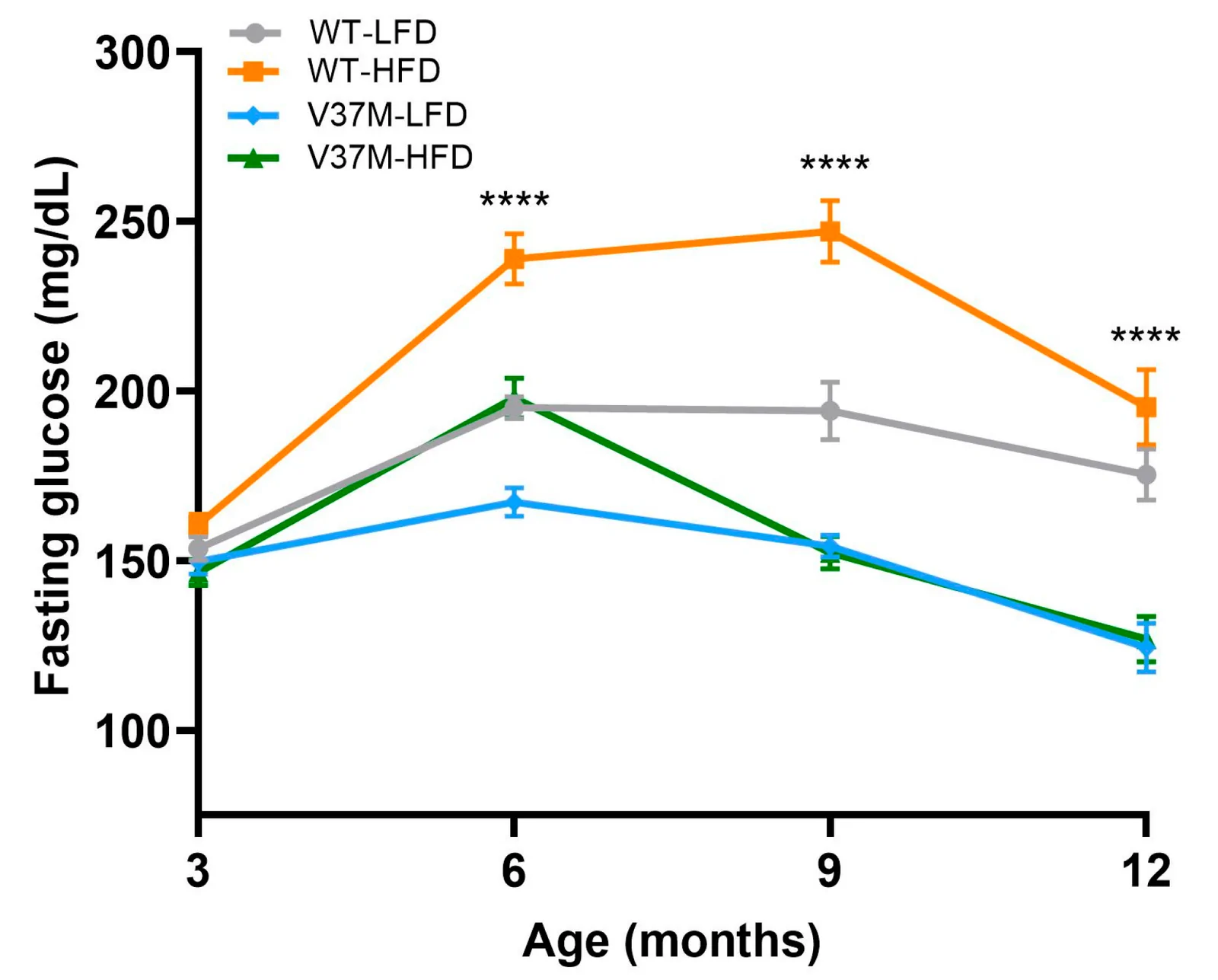
*Gjb2* V37M attenuates HFD-induced increases in fasting blood glucose. Fasting blood glucose levels were measured in male WT and homozygous *Gjb2* V37M mice fed a low-fat diet (LFD) or high-fat diet (HFD) at 3, 6, 9, and 12 months of age. Measurements at 3 months were obtained before initiation of the dietary intervention. Data are presented as mean ± SEM. Differences among the four groups at each age were analyzed using one-way ANOVA followed by Tukey’s multiple-comparisons test. Overall differences among groups were significant at 3 months (*p* = 0.0389) and at 6, 9, and 12 months (all *p* < 0.0001). Asterisks indicate the comparison between WT-HFD and V37M-HFD mice at the indicated time points (**** *p* < 0.0001). Abbreviations: WT, wild-type; LFD, low-fat diet; HFD, high-fat diet; SEM, standard error of the mean.

**Figure 3:**
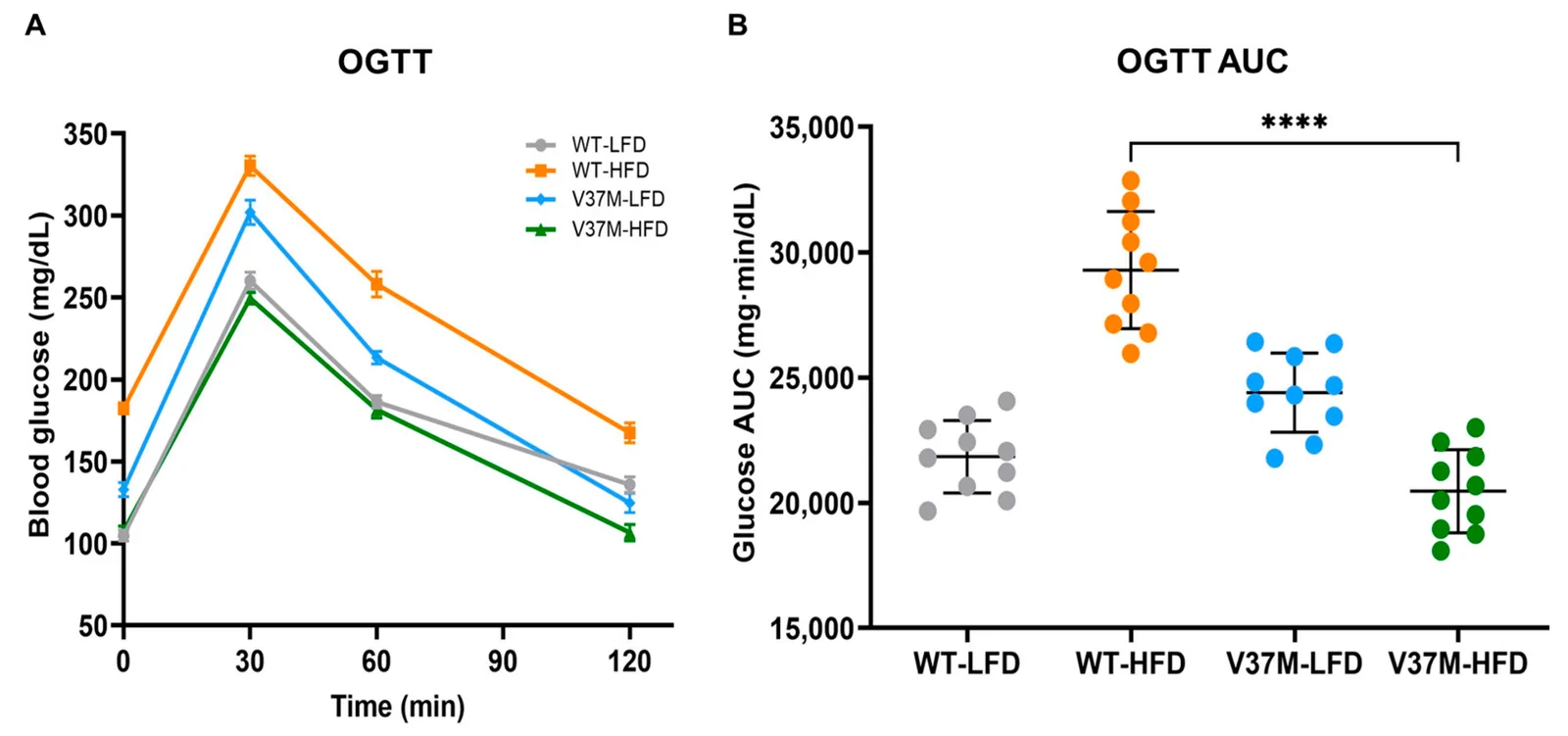
Gjb2 V37M modifies glucose responses during HFD feeding. (**A**) Oral glucose tolerance test (OGTT) performed at 9 months of age in male WT and homozygous *Gjb2* V37M mice fed a low-fat diet (LFD) or high-fat diet (HFD). Blood glucose concentrations were measured at 0, 30, 60, and 120 min after glucose administration. Data are presented as mean ± SEM. (**B**) Area under the glucose concentration–time curve from 0 to 120 min (AUC0–120), calculated from the OGTT glucose-response curves. Individual points represent individual mice, and horizontal lines with error bars indicate mean ± SD. Differences in AUC0–120 among the four groups were analyzed using one-way ANOVA followed by Tukey’s multiple-comparisons test (overall *p* < 0.0001). The bracket indicates the comparison between WT-HFD and V37M-HFD mice (**** *p* < 0.0001). Abbreviations: WT, wild-type; LFD, low-fat diet; HFD, high-fat diet; OGTT, oral glucose tolerance test; AUC, area under the curve; SEM, standard error of the mean; SD, standard deviation.

**Figure 4:**
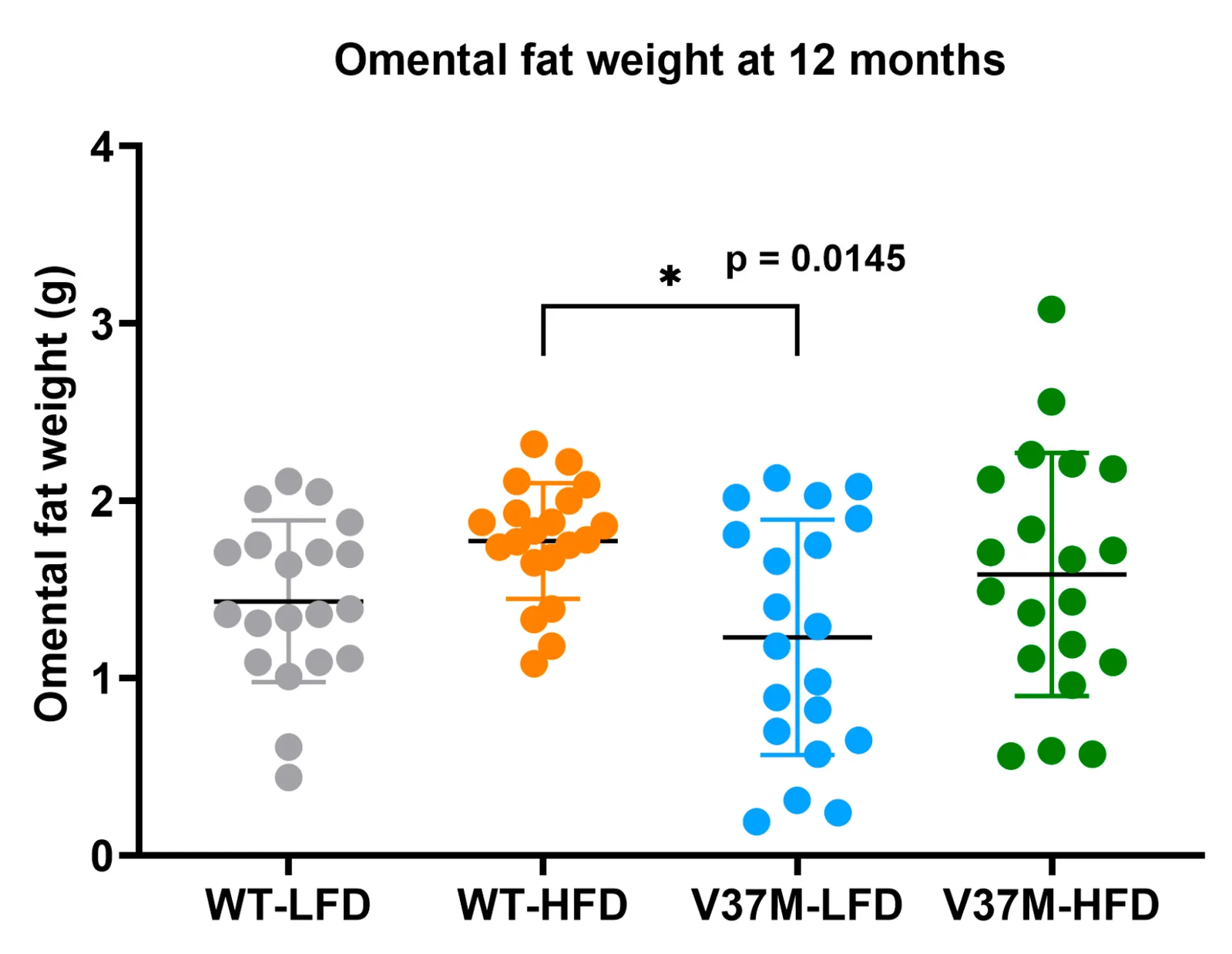
Omental fat weight at 12 months after chronic (9-month) HFD feeding. Individual points represent individual mice, and horizontal lines with error bars indicate mean ± SD. Differences among the four groups were analyzed using one-way ANOVA followed by Tukey’s multiple-comparisons test. The overall group effect was significant (*p* = 0.0210). The bracket indicates the significant pairwise comparison between WT-HFD and V37M-LFD mice (*p* = 0.0145). * *p* < 0.05. The sample size was *n* = 20 mice per group. Abbreviations: WT, wild-type; LFD, low-fat diet; HFD, high-fat diet; SD, standard deviation.

**Table 1:** Genotype × diet interaction effects on metabolic outcomes.

Outcome	Age	Interaction Effect *	95% CI	*p*-Value
Body weight (g)	6 mo	−7.50	−10.65 to −4.35	<0.0001
	9 mo	−21.80	−26.71 to −16.89	<0.0001
	12 mo	−21.60	−27.21 to −15.99	<0.0001
BMI (g/cm^2^)	6 mo	−0.91	−1.15 to −0.67	<0.0001
	9 mo	−1.33	−1.63 to −1.03	<0.0001
	12 mo	−1.32	−1.77 to −0.87	<0.0001
Triglycerides (mg/dL)	6 mo	−21.80	−34.25 to −9.35	0.0007
	9 mo	−3.90	−21.97 to 14.17	0.670
	12 mo	−25.00	−42.81 to −7.19	0.0063
HDL cholesterol (mg/dL)	6 mo	−13.90	−28.52 to 0.72	0.062
	9 mo	−27.60	−50.42 to −4.78	0.018
	12 mo	−25.50	−50.20 to −0.80	0.043
Fasting glucose (mg/dL)	6 mo	−13.30	−34.55 to 7.95	0.218
	9 mo	−54.70	−81.49 to −27.91	<0.0001
	12 mo	−17.40	−50.21 to 15.41	0.296

* The genotype × diet interaction effect was calculated as (HFD − LFD in V37M mice) − (HFD − LFD in WT mice). Negative values indicate a smaller HFD-associated increase in V37M mice than in WT mice. CI, confidence interval; BMI, body mass index; HDL, high-density lipoprotein cholesterol. Measurements at 3 months were obtained before initiation of the dietary intervention and are therefore not included in the post-intervention interaction table.

**Table 2:** Serum triglyceride and HDL cholesterol levels in WT and Gjb2 V37M mice during follow-up.

Variable	Time	WT-LFD	WT-HFD	V37M-LFD	V37M-HFD	*p*-Value *
Triglycerides, mg/dL	3 mo	45.6 ± 3.2	49.8 ± 8.5	66.4 ± 20.8	81.1 ± 44.4	<0.0001
	6 mo	43.1 ± 6.6	67.1 ± 17.3	59.5 ± 20.6	61.7 ± 27.9	0.0081
	9 mo	47.9 ± 9.2	58.3 ± 19.0	74.6 ± 36.2	81.1 ± 36.2	0.0011
	12 m	41.0 ± 13.9	59.1 ± 32.7	65.8 ± 18.8	58.9 ± 35.1	0.0027
HDL cholesterol, mg/dL	3 mo	43.8 ± 3.6	49.5 ± 5.1	78.5 ± 18.1	97.0 ± 14.8	<0.0001
	6 mo	61.1 ± 13.0	85.3 ± 33.8	72.6 ± 11.9	82.9 ± 26.6	0.0059
	9 mo	64.7 ± 19.8	106.9 ± 57.4	77.4 ± 16.6	92.0 ± 32.4	0.0003
	12 mo	81.1 ± 24.8	121.1 ± 61.5	66.9 ± 21.3	81.4 ± 24.0	<0.0001

Data are presented as mean ± SD. * *p*-values represent overall differences among the four groups by one-way ANOVA. WT, wild-type; LFD, low-fat diet; HFD, high-fat diet; HDL, high-density lipoprotein cholesterol; SD, standard deviation.

**Table 3:** Comparison of the composition and nutritional characteristics of the low-fat/reference diet and high-fat diet used in the study.

Characteristic	Low-Fat Diet (LFD)	High-Fat Diet (HFD)
Diet type	Standard rodent diet	Purified high-fat diet (D12492, Research Diets, Inc.)
Main ingredients	Corn, wheat, soybean meal, chicken meal, yeast powder, molasses, soybean oil	Casein, maltodextrin, sucrose, cellulose, soybean oil, lard
Protein	Crude protein ≥ 23%	20% of total calories; casein as the principal protein source
Fat content	Crude fat ≥ 4.5% by weight (approximately 12–13% of total energy from fat)	60% of total calories from fat
Major fat source	Soybean oil	Lard and soybean oil
Fiber	Crude fiber ≤ 5–6%	Cellulose
Feeding regimen	Ad libitum	Ad libitum

Abbreviations: LFD, low-fat/reference diet; HFD, high-fat diet. The two diets differed in ingredient composition as well as fat content.

## Data Availability

The original contributions presented in the study are included in the article. Further inquiries can be directed to the corresponding author.

## References

[1] Collins S., Martin T.L., Surwit R.S., Robidoux J. (2004). Genetic vulnerability to diet-induced obesity in the C57BL/6J mouse: Physiological and molecular characteristics. Physiol. Behav..

[2] d Oliveira R.B., Carvalho C.P.F., Polo C.C., Dorighello G.G.G., Boschero A.C., d Oliveira H.C.F., Collares-Buzato C.B. (2014). Impaired compensatory beta-cell function and growth in response to high-fat diet in LDL receptor knockout mice. Int. J. Exp. Pathol..

[3] West D.B., York B. (1998). Dietary fat, genetic predisposition, and obesity: Lessons from animal models. Am. J. Clin. Nutr..

[4] Mercer S.W., Trayhurn P. (1987). Effect of high fat diets on energy balance and thermogenesis in brown adipose tissue of lean and genetically obese ob/ob mice. J. Nutr..

[5] Jiang C.-L., Tsao C.-Y., Lee Y.-C. (2022). Vitamin C attenuates predisposition to high-fat diet-induced metabolic dysregulation in GLUT10-deficient mouse model. Genes Nutr..

[6] Paalvast Y., Zhou E., Rozendaal Y.J.W., Wang Y., Gerding A., van Dijk T.H., de Boer J.F., Rensen P.C.N., van Dijk K.W., Kuivenhoven J.A. (2022). A Systems Analysis of Phenotype Heterogeneity in APOE*3Leiden.CETP Mice Induced by Long-Term High-Fat High-Cholesterol Diet Feeding. Nutrients.

[7] Kadowaki T., Hara K., Kubota N., Tobe K., Terauchi Y., Yamauchi T., Eto K., Kadowaki H., Noda M., Hagura R. (2002). The role of PPARgamma in high-fat diet-induced obesity and insulin resistance. J. Diabetes Complicat..

[8] Kim E., Kim E.J., Seo S.W., Hur C.G., McGregor R.A., Choi M.S. (2014). Meta-review of protein network regulating obesity between validated obesity candidate genes in the white adipose tissue of high-fat diet-induced obese C57BL/6J mice. Crit. Rev. Food Sci. Nutr..

[9] Maestre I., Jordán J., Calvo S., Reig J.A., Ceña V., Soria B., Prentki M., Roche E. (2003). Mitochondrial dysfunction is involved in apoptosis induced by serum withdrawal and fatty acids in the beta-cell line INS-1. Endocrinology.

[10] Schaffer J.E. (2003). Lipotoxicity: When tissues overeat. Curr. Opin. Lipidol..

[11] Hamelin R., Allagnat F., Haefliger J.-A., Meda P. (2009). Connexins, diabetes and the metabolic syndrome. Curr. Protein Pept. Sci..

[12] Zhu Y., Chen H., Li D., Verkhratsky A., Yi C. (2026). Connexins in metabolic control: Linking brain cellular communication to systemic homeostasis. Life Med..

[13] Pérez-Armendariz E.M., Cruz-Miguel L., Coronel-Cruz C., Esparza-Aguilar M., Pinzon-Estrada E., Rancaño-Camacho E., Zacarias-Climaco G., Olivares P.F., Espinosa A.M., Becker I. (2012). Connexin 36 is expressed in beta and connexins 26 and 32 in acinar cells at the end of the secondary transition of mouse pancreatic development and increase during fetal and perinatal life. Anat. Rec..

[14] Carvalho C.P.F., Oliveira R.B., Britan A., Santos-Silva J.C., Boschero A.C., Meda P., Collares-Buzato C.B. (2012). Impaired β-cell–β-cell coupling mediated by Cx36 gap junctions in prediabetic mice. Am. J. Physiol. Endocrinol. Metab..

[15] Yanagiya T., Tanabe A., Hotta K. (2007). Gap-junctional communication is required for mitotic clonal expansion during adipogenesis. Obesity.

[16] Kim S.-N., Kwon H.-J., Im S.-W., Son Y.-H., Akindehin S., Jung Y.-S., Lee S.J., Rhyu I.J., Kim I.Y., Seong J.-K. (2017). Connexin 43 is required for the maintenance of mitochondrial integrity in brown adipose tissue. Sci. Rep..

[17] Guillebaud F., Barbot M., Barbouche R., Brézun J.-M., Poirot K., Vasile F., Lebrun B., Rouach N., Dallaporta M., Gaige S. (2020). Blockade of glial connexin 43 hemichannels reduces food intake. Cells.

[18] Santolim L.V., do Amaral M.E.C., Fachi J.L., Mendes M.F., de Oliveira C.A. (2017). Vitamin E and caloric restriction promote hepatic homeostasis through expression of connexin 26, N-cadherin, E-cadherin and cholesterol metabolism genes. J. Nutr. Biochem..

[19] Tirosh A., Tuncman G., Calay E.S., Rathaus M., Ron I., Tirosh A., Yalcin A., Lee Y.G., Livne R., Ron S. (2021). Intercellular transmission of hepatic ER stress in obesity disrupts systemic metabolism. Cell Metab..

[20] Yang Y.M., Seki E. (2021). Global spread of a local fire: Transmission of endoplasmic reticulum stress via connexin 43. Cell Metab..

[21] Naiki-Ito A. (2021). The roles of gap junctional intercellular communication in non-alcoholic steatohepatitis and liver fibrosis. Nihon Yakurigaku Zasshi.

[22] Batissoco A.C., Salazar-Silva R., Oiticica J., Bento R.F., Mingroni-Netto R.C., Haddad L.A. (2018). A cell junctional protein network associated with connexin-26. Int. J. Mol. Sci..

[23] Shen N., Peng J., Wang X., Zhu Y., Liu W., Liu A., Lu Y. (2017). Association between the p.V37I variant of *GJB2* and hearing loss: A pedigree and meta-analysis. Oncotarget.

[24] Tsai C.-Y., Lu Y.-C., Chan Y.-H., Radhakrishnan N., Chang Y.-Y., Lin S.-W., Liu T.-C., Hsu C.-J., Chen P.-L., Yang L.-W. (2023). Simulation-predicted and -explained inheritance model of pathogenicity confirmed by transgenic mice models. Comput. Struct. Biotechnol. J..

[25] Lin X., Li G., Zhang Y., Zhao J., Lu J., Gao Y., Liu H., Li G.-L., Yang T., Song L. (2019). Hearing consequences in *Gjb2* knock-in mice: Implications for human p.V37I mutation. Aging.

[26] Fetoni A.R., Zorzi V., Paciello F., Ziraldo G., Peres C., Raspa M., Scavizzi F., Salvatore A.M., Crispino G., Tognola G. (2018). Cx26 partial loss causes accelerated presbycusis by redox imbalance and dysregulation of Nrf2 pathway. Redox Biol..

[27] Martínez A.D., Acuña R., Figueroa V., Maripillan J., Nicholson B. (2009). Gap-junction channels dysfunction in deafness and hearing loss. Antioxid. Redox Signal..

[28] Ke Y., Liu X., Sun Y. (2025). Regulatory mechanisms of connexin26. Neuroscience.

[29] Wu X., Wang Y., Sun Y., Chen S., Zhang S., Shen L., Huang X., Lin X., Kong W. (2014). Reduced expression of connexin 26 and its DNA promoter hypermethylation in the inner ear of mimetic aging rats induced by D-galactose. Biochem. Biophys. Res. Commun..

[30] Hwang J.H., Wu C.C., Hsu C.J., Liu T.C., Yang W.S. (2009). Association of central obesity with the severity and audiometric configurations of age-related hearing impairment. Obesity.

[31] Hwang J.H., Hsu C.J., Liu T.C., Yang W.S. (2011). Association of plasma adiponectin levels with hearing thresholds in adults. Clin. Endocrinol..

[32] Hwang J.H. (2017). Effects of obesity on protein kinase C, brain creatine kinase, transcription, and autophagy in cochlea. Metab. Brain Dis..

[33] Lee S.-Y., Fontana F., Sugatani T., Portales Castillo I., Leanza G., Coler-Reilly A., Civitelli R. (2024). Connexin43 in mesenchymal lineage cells regulates body adiposity and energy metabolism in mice. JCI Insight.

[34] Fu J., Liu S., Li M., Guo F., Wu X., Hu J., Wen L., Wang J., Li X. (2024). Optimal fasting duration for mice as assessed by metabolic status. Sci. Rep..

[35] Ayala J.E., Samuel V.T., Morton G.J., Obici S., Croniger C.M., Shulman G.I., Wasserman D.H., McGuinness O.P., NIH Mouse Metabolic Phenotyping Center Consortium (2010). Standard operating procedures for describing and performing metabolic tests of glucose homeostasis in mice. Dis. Model. Mech..

[36] Brundert M., Heeren J., Merkel M., Carambia A., Herkel J., Groitl P., Dobner T., Ramakrishnan R., Moore K.J., Rinninger F. (2011). Scavenger receptor CD36 mediates uptake of high density lipoproteins in mice and by cultured cells. J. Lipid Res..

[37] Rinninger F., Heine M., Singaraja R., Hayden M., Brundert M., Ramakrishnan R., Heeren J. (2014). High density lipoprotein metabolism in low density lipoprotein receptor-deficient mice. J. Lipid Res..

[38] Pellizzon M.A., Ricci M.R. (2018). The common use of improper control diets in diet-induced metabolic disease research confounds data interpretation: The fiber factor. Nutr. Metab..

[39] Ables G.P., Perrone C.E., Orentreich D., Orentreich N. (2012). Methionine-Restricted C57BL/6J Mice Are Resistant to Diet-Induced Obesity and Insulin Resistance but Have Low Bone Density. PLoS ONE.

